# Supplementary material for: Causal Relationship between Aging and Anorexia Nervosa: A White-Matter-Microstructure-Mediated Mendelian Randomization Analysis
Source: Biomedicines. 2024 Aug 16;12(8):1874. doi: 10.3390/biomedicines12081874 (PMC11351342; doi:10.3390/biomedicines12081874)
Supplement: Supplementary file 1 [file biomedicines-12-01874-s001.zip › Supplementary Figure/Figure S4/Singe SNP plot of MR analysis for TL to ubm-b-2126.pdf]

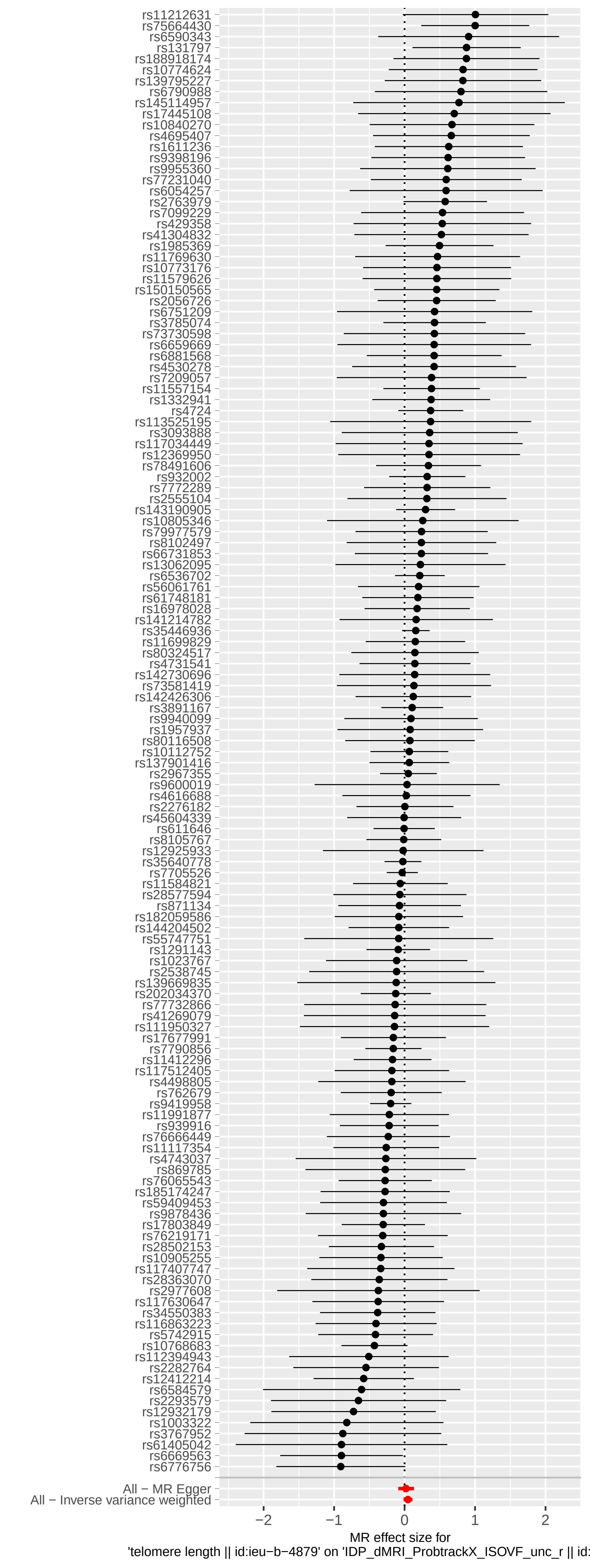

rs11212631
rs75664430
rs6590343
rs131797
rs188918174
rs10774624
rs139795227
rs6790988
rs145114957
rs17445108
rs10840270
rs4695407
rs1611236
rs9398196
rs9955360
rs77231040
rs6054257
rs2763979
rs7099229
rs429358
rs41304832
rs1985369
rs11769630
rs10773176
rs11579626
rs150150565
rs2056726
rs6751209
rs3785074
rs73730598
rs6659669
rs6881568
rs4530278
rs7209057
rs11557154
rs1332941
rs4724
rs113525195
rs3093888
rs117034449
rs12369950
rs78491606
rs932002
rs7772289
rs2555104
rs143190905
rs10805346
rs79977579
rs8102497
rs66731853
rs13062095
rs6536702
rs56061761
rs61748181
rs16978028
rs141214782
rs35446936
rs11699829
rs80324517
rs4731541
rs142730696
rs73581419
rs142426306
rs3891167
rs9940099
rs1957937
rs80116508
rs10112752
rs137901416
rs2967355
rs9600019
rs4616688
rs2276182
rs45604339
rs611646
rs8105767
rs12925933
rs35640778
rs7705526
rs11584821
rs28577594
rs871134
rs182059586
rs144204502
rs55747751
rs1291143
rs1023767
rs2538745
rs139669835
rs202034370
rs77732866
rs41269079
rs111950327
rs17677991
rs7790856
rs11412296
rs117512405
rs4498805
rs762679
rs9419958
rs11991877
rs939916
rs76666449
rs11117354
rs4743037
rs869785
rs76065543
rs185174247
rs59409453
rs9878436
rs17803849
rs76219171
rs28502153
rs10905255
rs117407747
rs28363070
rs2977608
rs117630647
rs34550383
rs116863223
rs5742915
rs10768683
rs112394943
rs2282764
rs12412214
rs6584579
rs2293579
rs12932179
rs1003322
rs3767952
rs61405042
rs6669563
rs6776756
All − MR Egger
All − Inverse variance weighted
−2
−1
0
1
2
MR effect size for
'telomere length || id:ieu−b−4879' on 'IDP_dMRI_ProbtrackX_ISOVF_unc_r || id:
